# A community-engaged randomized controlled trial assessing the effectiveness of a weight loss aid in reducing the body burden of persistent organic pollutants

**DOI:** 10.1016/j.envadv.2026.100742

**Published:** 2026-07-31

**Authors:** Metrecia L. Terrell, Martha Scott Tomlinson, Joshua Wolfe, Robert B. Hood, Melanie A. Pearson, Hillary Barton, Sehar Dadani, Jennifer Morse, Dana Boyd Barr, Bonnie Havlicek, Brittany B. Fremion, Jane Jelenek, Norman Keon, Thomas H. Corbett, Alexander H.K. Montoye, Terryl J. Hartman, Amita Manatunga, Michele Marcus

**Affiliations:** a Department of Epidemiology, Emory University Rollins School of Public Health, 1518 Clifton Rd. NE, Atlanta, GA 30322, USA; b Division of Epidemiology, Ohio State University College of Public Health, 1841 Neil Avenue, Columbus, OH 43210, USA; c Gangarosa Department of Environmental Health, Emory University Rollins School of Public Health, 1518 Clifton Rd. NE, Atlanta, GA 30322, USA; d Mid-Michigan District Health Department, 615 N State St., Stanton, MI 48888, USA; e PBB Citizens Advisory Board, Michigan, USA; f Department of History, World Languages & Cultures, Central Michigan University, 1200 S. Franklin St., Mount Pleasant, MI 48859, USA; g Pine River Superfund Citizen Task Force, P.O. Box 172, St. Louis, MI 48880, USA; h Exercise Science Program, Montcalm Community College, 2800 College Dr., Sidney, MI 48885, USA; i Department of Kinesiology, Michigan State University, 426 Auditorium Rd., East Lansing, MI 48824, USA; j Department of Biostatistics and Bioinformatics, Emory University Rollins School of Public Health, 1518 Clifton Rd. NE, Atlanta, GA, 30322, USA

**Keywords:** Polybrominated biphenyl, Persistent organic pollutants, Brominated flame retardants, Randomized controlled trial, Orlistat, Community-engaged research

## Abstract

**Background:**

Exposure to persistent organic pollutants (POPs) can impact human health and have multigenerational consequences. These chemicals are particularly concerning because they accumulate in adipose tissue and are eliminated slowly, leading to prolonged internal exposure and potential long-term health effects.

**Methods:**

To assess whether a weight loss aid could reduce the body burden of POPs, we conducted a community-engaged randomized, quadruple-blind, placebo-controlled trial in a population highly exposed to a brominated flame retardant, polybrominated biphenyl (PBB). Blood samples, anthropometric data, and other study assessments were collected at baseline, 3 months, and 6 months. The aim was to determine if orlistat use resulted in weight loss accompanied by a decrease in concentrations of PBB and other persistent chemicals (PBDEs, PCBs, and p,p’-DDE).

**Results:**

Of the 100 participants initially enrolled, 89 (placebo, n=45; orlistat, n=44) completed the six-month study. We found greater weight loss in the orlistat group, losing 2 kg more on average compared to the placebo group (Month 6 group difference: −1.99, 95% CI: −3.81, −0.18). POP models showed no statistically significant group differences, or a reduction in serum concentrations over the six months.

**Conclusions:**

This study began because community members raised questions about how to eliminate PBB from the body. Participants’ dedication was evident by the low attrition rate (11%). A six-month orlistat intervention did not result in lower serum concentrations of PBB or other POPs. Further research is needed to determine effective ways to decrease the body burden of this class of chemicals.

## 1. Introduction

Persistent organic pollutants (POPs) encompass a range of legacy chemicals that persist in the environment and can subsequently affect human health. The impact on the body is long-lasting, as these chemicals have long half-lives, sequestering into lipid-rich deposition matrices and biomagnifying in the food chain. They accumulate in and are slowly released from adipose tissue, which can cause a constant source of long-term exposure in the body (La Merrill et al., 2013). Effective methods for lowering POP concentrations in the body have not been established. Detoxification regimens and nutritional interventions have been reviewed, highlighting a growing need for and continuing interest in this research area (Corbett et al., 2022; Klein & Kiat, 2015; Sargis et al., 2019). According to an early review, introducing a nonabsorbable lipid into the diet could increase the excretion rate of lipophilic chemicals (Jandacek, 1982). A pilot study using a rodent model showed Olestra, a fat-substitute that was previously used in U.S. food products, increased the fecal excretion rate of organochlorine compounds (Jandacek & Tso, 2007; Jandacek et al., 2005). Human pilot studies of Olestra were also found to decrease concentrations of polychlorinated biphenyls (PCBs) (Jandacek et al., 2014) and an organochlorine pesticide (OCP), b-hexachlorocyclohexane (B-HCH) (Arguin et al., 2010). We proposed this randomized controlled trial in a population with high levels of polybrominated biphenyls (PBBs), a brominated flame retardant, because of the lack of studies assessing interventions to reduce the body burden of these types of chemicals.

In 1973, the Velsicol Chemical Corporation plant in St. Louis, Michigan, shipped a flame-retardant mixture containing PBBs instead of a nutritional supplement to Michigan Farm Bureau Services, where it was added to livestock feed and delivered to farms across Michigan. PBBs entered the food chain, ultimately exposing millions of Michigan residents who consumed contaminated dairy and animal products (Fries, 1985; Landrigan PJ et al., 1979). Contaminated products remained in the food supply until around 1977 (Corbett, 2023). To evaluate the long-term health effects of PBB exposure, the Michigan Department of Public Health, now the Michigan Department of Health and Human Services, with support from the U.S. Public Health Service and multiple federal partners, established the Michigan Long-Term PBB Study in 1976. Today, the study is known as the Michigan PBB Registry, managed by Emory University, and is a multigenerational cohort with over 7,500 individuals. The PBB Registry includes those directly exposed during the contamination incident and subsequent generations, as PBB is transferred from mother to child across the placenta and through breastfeeding (Joseph et al., 2009). Health-related research has been ongoing with this cohort for over 50 years, and associations between PBB exposure and various adverse health effects have been documented (Hoffman et al., 2025). Analysis of blood samples from registry members from 2012 to 2015 showed that approximately 60% of those tested still had elevated blood levels of PBB-153 (Chang et al., 2020), the main PBB congener detected in humans, when compared to the 95^th^ percentile in the general population (U.S. National Health and Nutrition Examination Survey, NHANES). PBB-153, like other POPs, has a long half-life in humans (median about 12 years) and is slowly eliminated from the body (Hood et al., 2023).

The intervention in this study, orlistat, is available in prescription and over-the-counter (OTC) formulations. It is a weight loss product approved by the U.S. Food and Drug Administration (FDA) in 2007 (U.S. Food and Drug Administration, 2015), intended for use with a reduced-calorie, low-fat diet. Orlistat is a lipase inhibitor that helps break down dietary fats for absorption in the intestines. It has been shown to reduce the absorption of dietary fats by 30% and increase fecal fat excretion (McClendon et al., 2009; Zhi et al., 1994). Experimental studies of orlistat suggest it may act similarly to Olestra by reducing reabsorption and enhancing elimination of persistent chemicals (Jandacek & Tso, 2007; Jandacek et al., 2005). Because orlistat preferentially reduces fat absorption, its presence could contribute to eliminating fat-soluble chemicals from the body (Jandacek & Genuis, 2013). With weight loss, a shift in the equilibrium of lipophilic chemicals from lipid-rich matrices to the blood may temporarily increase blood levels but also allow them to bind to intestinal fat that is excreted into the feces.

The Michigan PBB Registry Clinical Trial originated from a 13-year partnership between researchers and community partners who were distressed to discover that over 40 years after the exposure incident, many individuals still had elevated levels of PBBs and were concerned about the ongoing effects of continued exposure. We designed and conducted this community-engaged clinical trial to investigate whether the use of orlistat (at the OTC dose) combined with diet and exercise for six months (enrollment, 3-month, and 6-month follow-up visits) results in the elimination of PBB and other similar POPs, as measured by changes in serum concentrations. Identifying an effective intervention to reduce the body’s burden of lipophilic toxicants is of paramount importance to the affected community, as well as to other communities with high POP exposures and members of the general population who are exposed to lipophilic chemicals daily, primarily through their diet.

## 2. Methods

### 2.1. Study Design and Participants

The Michigan PBB Registry Clinical Trial was a randomized, quadruple-blinded, placebo-controlled trial conducted from September 2018 to June 2022 among Michigan PBB Registry participants who were recruited and enrolled in waves during this period. The study was registered with clinicaltrials.gov (NCT03582722). The Emory Institutional Review Board approved the study protocol, and all participants provided informed consent.

Participants were recruited primarily at community meetings statewide. We mailed invitations and infographics describing the study before and after the meetings. Study eligibility was determined through a two-step process: 1) a health screening questionnaire and 2) clinical bloodwork results collected at a baseline appointment. Trained study staff from the Emory research team conducted the initial health screening questionnaire over the phone or in person. The screening included general eligibility and health questions, which were later reviewed by the study physician (JM). Verbal consent was obtained before administering the health screening questionnaire. Individuals meeting the initial inclusion criteria and who did not have any of the initial exclusion criteria were deemed provisionally eligible for the study (Table 1). Individuals with Crohn’s disease, ulcerative colitis, celiac disease, diabetes, thyroid disorder, or undiagnosed abdominal pain or diarrhea were only eligible following consultation with and approval from the study physician and/or their primary care provider.

If deemed provisionally eligible, participants provided informed consent and scheduled a baseline appointment held at local health department facilities. Blood samples were collected for analysis of environmental chemicals, including PBB-153, polybrominated diphenyl ethers (PBDEs), PCBs, dichlorodiphenyltrichloroethane (DDT) and dichlorodiphenyldichloroethylene (DDE). Analysis focused on specific congeners that are the major components of the products or the most frequently detected congeners in the environment (Darrow et al., 2017; IARC, 2016). In addition, blood samples were analyzed for clinical tests, including a lipid panel, creatinine with estimated glomerular filtration rate (eGFR; kidney function), alanine aminotransferase (ALT; liver function), and thyroid-stimulating hormone (TSH; thyroid function). The study physician reviewed clinical laboratory results to determine final eligibility. Participants were ineligible for the study if they had abnormal eGFR or ALT levels. We informed participants of their clinical test results as soon as they became available, and the study physician was available to answer any questions they might have had about the test results.

### 2.2. Randomization and Blinding

Participants were randomized into two groups: the intervention group (orlistat) and the control group (placebo). Participant assignments were stratified by sex (assigned at birth) and body mass index (BMI) (25.0–29.9 kg/m^2^ and ≥30.0 kg/m^2^). A randomization plan was generated using a pseudo-random number generator with randomly permuted blocks to balance group assignments throughout the study. Permuted block sizes were not disclosed to the blinded study personnel to minimize the likelihood of their being able to predict the next randomization assignment in the series. These assignments were securely stored, with access limited to the lead biostatistician (AM), who informed the pharmacy of the group assignment using a unique subject-specific identification number composed of abbreviations for BMI status and sex stratum. The study was quadruple-blind; neither investigators, study coaches, outcome assessors, nor participants were informed of the group assignments. Data cleaning and statistical analyses were also performed by blinded study personnel (MLT) until completion.

### 2.3. Study Intervention

The intervention group was instructed to take one 60 mg capsule of orlistat and the control group a placebo capsule with each meal containing fat, up to three capsules daily, for six months. Both groups received the same daily multivitamin-mineral supplement (Ingredient Lists in Supplemental Table S1) and were prescribed the same diet and exercise regimen, as well as a weight management intervention. Participants were assigned study coaches and had weekly phone calls with their coaches for the first three months, followed by biweekly phone calls until the trial concluded at six months. The coaches were blind to participants’ group assignments. During the weekly phone calls, coaches conducted lessons on diet, weight loss, and weight management and set goals with the participants.

### 2.4. Weight management intervention

The weight management intervention was designed and supervised by a registered dietitian and nutrition epidemiologist (TJH). The study coaches discussed strategies and supported the participants in lowering dietary fat intake and increasing physical activity. Both study groups’ weight loss and management strategies were consistent with the FDA-approved patient information for orlistat that recommends limiting overall dietary fat intake to 30% of daily energy, with energy intake adjusted to lose 0.5–1 kg per week (FDA-Approved Weight Loss Aid | Alli^®^).

Participants in both groups received guidance on nutrition, including lessons on reading food labels, determining appropriate portion sizes, reducing overall dietary fat content, replacing higher energy-dense foods with lower energy-dense options such as fruits, vegetables, and whole grains, meal planning, and grocery shopping. All information was provided as behaviorally-based strategies similar to those used in the Diabetes Prevention Program (Diabetes Prevention Program Research, 2002) and Look Action for Health in Diabetes (Look Ahead Research Group, 2006) lifestyle intervention programs (Foster-Schubert et al., 2012; Mason et al., 2011) that used social cognitive theory (Bandura, 2002).

### 2.5. Study Assessments

The data collected for the study were stored in REDCap (Harris et al., 2019; Harris et al., 2009). Anthropometric measures, diet assessments, physical activity assessments, and blood draws were conducted at baseline (or enrollment), three months, and six months (trial conclusion) (Supplemental Table S2). We implemented study modifications during the COVID-19 pandemic. This group of participants (n=23) could not attend the three-month study visits because of restrictions and safety concerns. They did not have a three-month blood sample collected, but they completed the three-month diet and physical activity assessments and self-reported their weight (using a home scale) and waist circumference (with the provided Myo-tapes and training videos).

#### 2.5.1. Blood collection and laboratory assessments

Non-fasting blood samples were collected in serum-separator tubes (SST) at each study visit. Samples were allowed to sit at room temperature for 30 minutes to allow time for clotting. Samples were stored on wet ice or refrigerated until processed within a 72-hour window. At the time of processing, the tubes were spun to isolate the serum. The serum was immediately aliquoted into 2 mL cryovials and stored at −80°C at Emory University until analyzed for exposure concentrations. Additional blood samples were collected at each study visit in SSTs and sent to Quest Diagnostics for clinical testing. Quest Diagnostics analyzed the baseline and six-month blood samples for a lipid panel, creatinine, eGFR, ALT, and TSH tests. The three-month testing included only the lipid panel.

Laboratory analyses of serum samples for exposure concentrations were conducted at Emory University’s Laboratory for Exposure Assessment and Development in Environmental Research (LEADER). Samples from all participants were analyzed at the end of the study to limit batch-to-batch variability. Each participant’s samples were also analyzed in the same batch (in random study visit order) to limit variability. Samples were analyzed for the following targeted congeners: PBB-153, PCBs (118, 138, 153, 180) using the methods described in Marder et al. (2016); DDT (as p,p’-DDT) and DDE as (p,p’-DDE) using the methods described in Gaspar et al. (2017); and PBDEs (47, 99, 100), using the methods described in Chang et al. (2020). The limit of detections (LODs) were 0.01 ng/mL for PBB-153, 0.02 ng/mL for the PCB congeners, DDT and DDE, 0.0025 ng/mL for PBDE-47, and 0.0125 ng/mL for PBDE-99 and PBDE-100. Relative standard deviations of the analytes were <12%. We excluded chemicals with low detection rates (for PCB-118, PBDE-99, PBDE-100, and DDT), and we summed the di-ortho chlorinated PCB congeners (PCB-138, PCB-153, and PCB-180) to represent total PCB (∑PCB_3_). We calculated total serum lipids using the method published by Phillips et al. (Phillips et al., 1989).

#### 2.5.2. Anthropometric measurements

Anthropometric measurements were conducted at each study visit by study staff and taken in triplicate following procedures outlined in the NHANES Anthropometry Procedures Manual (Centers for Disease Control and Prevention, 2016). Participants had their weight, body fat percentage, and muscle mass percentage measured in light clothing without shoes or socks using the same bioimpedance scale at each visit (BF525 Glass Body Analysis Scale, Beurer). Height was measured using a stadiometer. A cloth measuring tape (or MyoTape body measure tape) was placed horizontally just above the iliac crest after completion of a normal expiration to measure waist circumference.

#### 2.5.3. Dietary assessments

We assessed recall of food and beverage intake using the Automated Self-administered 24-hour Recall (ASA24) (Frankenfeld et al., 2012; Subar et al., 2012). A 24-hour recall was used because it is a preferred method for measuring short-term changes in food intake, providing high-quality and relatively unbiased dietary data (Thompson & Subar, 2017). During the week before randomization, two unannounced 24-hour recall emails were sent to participants, one on a weekday and one on a weekend (to log their diet from approximately midnight to midnight of the preceding day). This process was repeated during the six-month assessment. Participants received only one request to recount their diet in month 3. The ASA24 data were used to calculate the Healthy Eating Index (HEI-2015), a measure of diet quality (Krebs-Smith et al., 2018).

#### 2.5.4. Physical activity assessments

Physical activity was assessed at each time point and across several domains (leisure, domestic and yard work, and transportation-related activities) to evaluate if any changes in activity level explained the differences in weight loss between study groups. We developed the Physical Activity Questionnaire (PAQ) by adapting the Women’s Health Initiative Physical Activity Questionnaire (Women’s Health Initiative Physical Activity Questionnaire, 2015) for use by men and by incorporating additional activities typical of rural Michiganders (e.g., hunting, cross-country skiing, and bowling) suggested by our community partners. We used PAQ data to create measures of exercise intensity (moderate and strenuous, minutes per week) and to calculate a total physical activity score (MET-hours per week) (Meyer et al., 2009).

### 2.6. Safety and adherence

Coaches inquired about adverse events (AEs) during phone calls. In addition, we systematically tracked gastrointestinal symptoms at each study time point with the Gastrointestinal Symptom Tracker (GST) adapted from the AbbVie Exocrine Pancreatic Insufficiency GI Symptom Tracker (AbbVie Inc). The GST captured the frequency of specific symptoms that are known side effects of the intervention. AEs were recorded by coaches and reviewed by the study team, including the Principal Investigator (MM) and study physician, as required. Coaches called participants one month after the trial’s completion for a seven-month phone check-in, addressing final questions and following up on any ongoing adverse events. We classified AEs as serious or non-serious events. Non-serious events were classified by condition type and specific gastrointestinal disorders. Coaches collected data to assess adherence to the protocol during phone calls. Participants were asked how many days (in the past week) they had taken their study medication and how many pills they were taking each day. In addition, participants were asked to report pill counts (or estimates of remaining pills) in months 3 and 6. Adherence was determined based on consistent study medication usage (~80% taken over 6 months) and completion of other study components (Supplemental Table S3).

### 2.7. Statistical analyses

#### 2.7.1. Outcome measures

We had two study outcomes. The primary outcome was the change in body weight in the orlistat group compared to the placebo group from enrollment to six months. The secondary outcome was the change in serum POP concentrations in the orlistat group compared to the placebo group from enrollment to six months.

#### 2.7.2. Sample size

The power of the study was calculated based on the study outcomes of changes in body weight or serum POP concentrations. Previous studies of orlistat have shown an average weight difference of 2 to 3 kg between the intervention and placebo groups (Li et al., 2005). Assuming a standard deviation of 5 kg for weight change, we would have adequate power (80%) to detect a 2 to 3 kg difference (α =0.05; ρ _=_0.5) in weight, which is considered clinically relevant (Blackburn, 1995), with a sample size of 50 in each group. For changes in serum POP concentrations, we based our sample size estimations on prior data from PBB Registry participants with at least two PBB samples collected within a year of each other, and the pilot data from Jandacek et al.’s study (Jandacek et al., 2014). We estimated that the variance of the differences in serum PBB levels was expected to be between 0.1 and 0.2 log ng/mL using the pooled variances for our power calculations. We expected a 1% reduction in PBB levels because of the natural course of exposure. A sample size of 50 in each group would provide at least 80% power to detect a mean change in log PBB levels of −0.1 and −0.2 log ng/mL with a significance level of α =0.05. Power analyses were performed in PASS 13 software (PASS 13 Power Analysis and Sample Size Software, 2014).

#### 2.7.3. Statistical methods

We explored baseline characteristics and changes in body weight, POP concentrations, and other assessments across study groups. We examined longitudinal trends with individual plots and plots of mean POP concentrations over time by group. To explore the relationship between weight loss and POP concentrations, we generated Spearman’s correlation statistics and LOESS curves for changes in body weight by changes in POP concentrations. The data were analyzed according to the intent-to-treat principle. We used constrained linear data analysis models (cLDA) to assume a common baseline mean across study groups and to account for repeated measures (Liang & Zeger, 2000). Model estimates were presented as least squares mean changes and differences, along with corresponding 95% confidence intervals (CI). The models were adjusted for the stratification variables (sex and BMI) and included a group variable (orlistat or placebo), study time (0, month 3, and month 6), and their interaction (group × time) as fixed effects. The interaction term was included to determine if the mean change in outcomes varied by group. We used an unstructured covariance structure for study time.

The POP concentrations were natural log transformed (ln-transformed) because of their right-skewness, with concentrations below the LOD imputed as LOD/√2 before transformation (Hornung & Reed, 1990). Model estimates from ln-transformed exposures can be expressed as the percentage change in concentrations, calculated as (e^β^−1) × 100. We assessed POP concentrations over time on a wet weight basis (ng/mL). Some models were adjusted for total serum lipids (as a continuous variable), or the POPs were standardized for lipids (ng/g lipid) (Brown & Lawton, 1984; Schisterman et al., 2005). In secondary models, we adjusted for several additional covariates, including study age, diet quality, and intensity of physical activity. We performed analyses to assess differences in exposure concentrations within two sub-groups of interest: 1) those with the highest POP concentrations at baseline (≥75^th^ percentile); and 2) those who lost the most body weight during the study (weight loss ≥5% baseline weight). All statistical analyses and model diagnostics were performed in SAS 9.4 software, and models were fitted with the Mixed procedure (SAS Institute Inc., 2016).

## 3. Results

### 3.1. Study participants

**Fig. 1.**
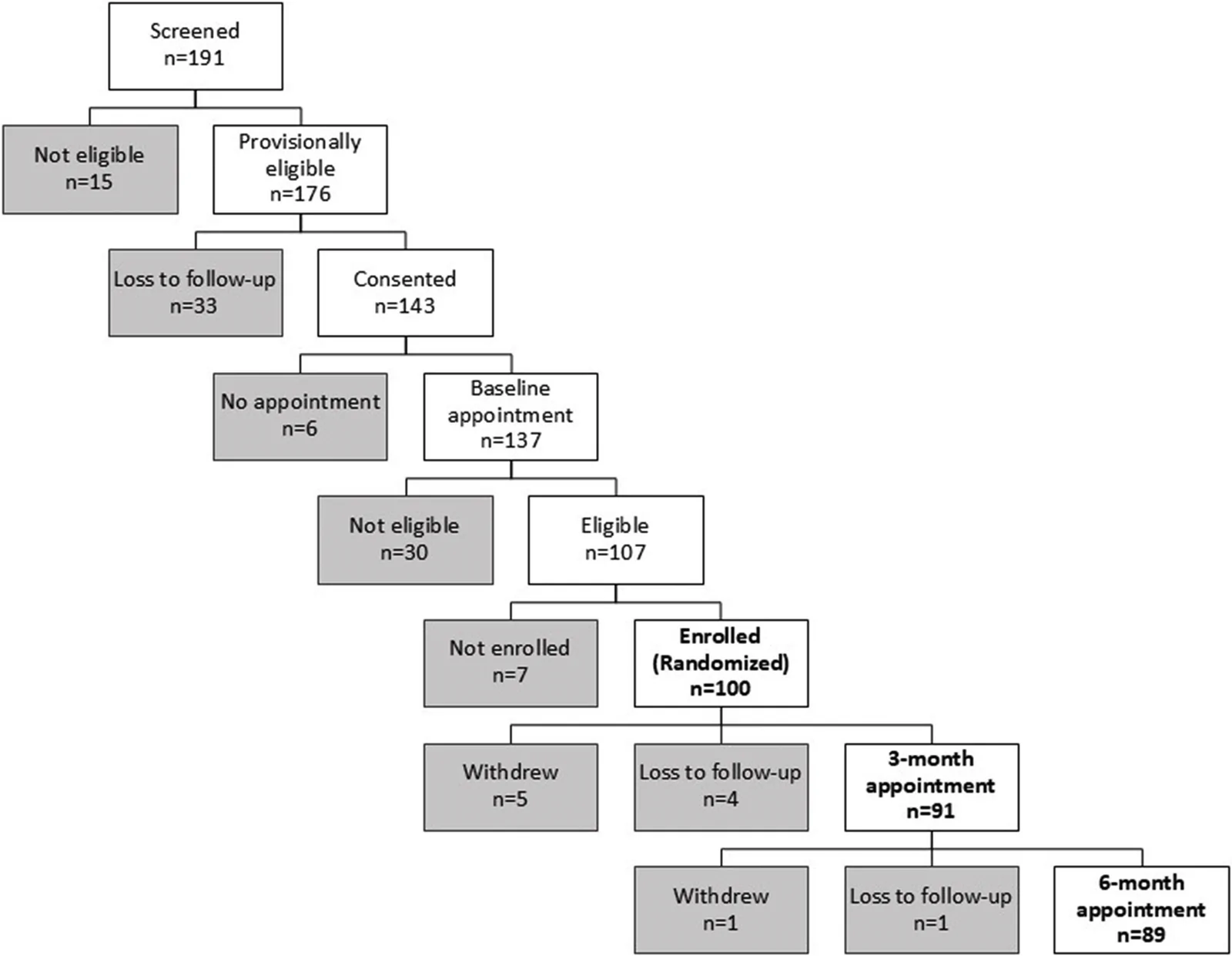
Michigan PBB registry clinical trial - screening, eligibility and study numbers.

We screened 191 individuals, and 176 (92%) were considered provisionally eligible based on their answers to the health screening questions and review by the study physician (Fig. 1). Of these, 33 did not consent to further participation, leaving 143 participants eligible for a baseline appointment; of whom 137 (96%) completed the appointment. From the baseline clinical laboratory results, 29 (21%) were ineligible because of abnormal eGFR or ALT levels, and one was ineligible because of incomplete clinical laboratory results. Before randomization, seven individuals decided they no longer wanted to participate, resulting in 100 participants who were enrolled and randomized.

Of the 100 enrolled participants, 89 completed the six-month study (placebo group, n=45; orlistat group, n=44). Within the first three months of study participation, five participants withdrew, and four were lost to follow-up. Reasons for withdrawal included stress related to COVID-19, negative effects from the study medication, or other personal reasons. Following the three-month study visit, we had one withdrawal because of other medical concerns and one lost to follow-up (Fig. 1). All 89 participants had baseline and six-month blood draws, and 65 participants had three-month blood draws (23 missing because of the COVID-19 pandemic and 1 missing for other reasons).

### 3.2. Demographic and baseline characteristics

Study groups had similar demographics, comprising mostly females (60%) and who were predominantly white (94%). Participants’ median study age was 60 years (Table 2). The median baseline body weight was 90.5 kg [77.8, 103.3] among participants in the placebo group and 88.7 kg [80.8, 104.9] in the orlistat group. In the placebo group, 62.2% were classified as obese, and 56.8% were classified as obese in the orlistat group. At baseline, participants had a median total cholesterol level of 187 mg/dL [162, 217] in the placebo group and 210.5 mg/dL [184, 239] in the orlistat group.

At enrollment, dietary data were comparable across study groups. Overall, the total fat intake was 38% kcal, saturated fat intake was 12.5% kcal, and fiber intake was 8.7 g/1000 kcal (Table 2). Compared to the orlistat group, the placebo group had a lower physical activity intensity score (placebo group: 8.3 MET-hours/week; orlistat group: 13.5 MET-hours/week). The overall median serum PBB-153 concentration at baseline was 0.351 ng/mL, with the placebo group median (0.469 ng/mL) slightly higher than in the orlistat group (0.260 ng/mL). Serum concentrations of the other chemicals at baseline were more similar between the study groups (overall medians: PBDE-47, 0.030 ng/mL; ΣPCB_3_, 0.285 ng/mL; DDE, 0.518 ng/mL) (Table 2).

### 3.3. Primary outcome: changes in body weight

**Fig. 2.**
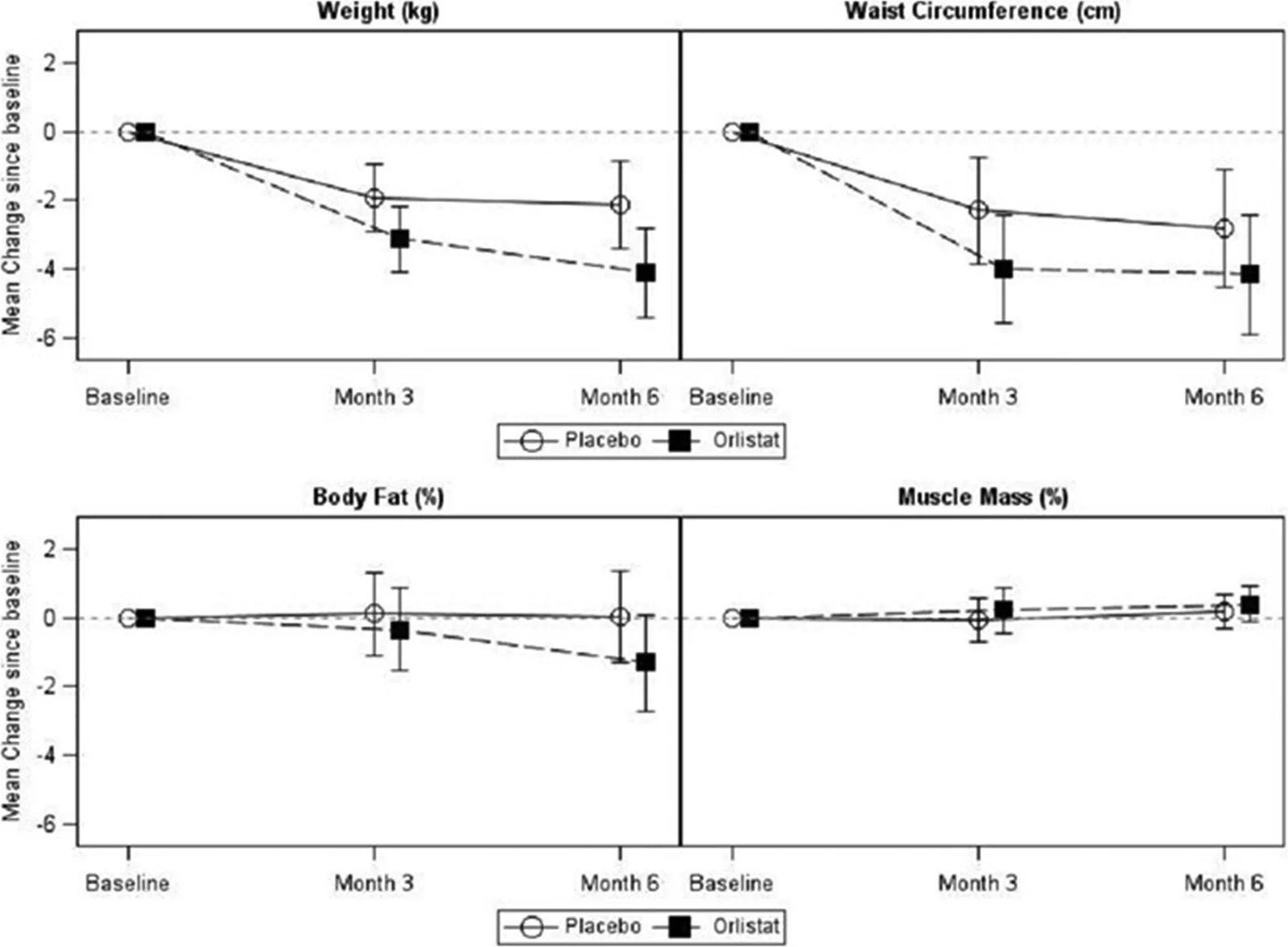
Michigan PBB registry clinical trial - mean change from baseline for anthropometric measures. estimates and 95% Cls from constrained linear data analysis models adjusted for stratification variables (sex and BMI).

Analysis of the primary outcome showed a significant between-group difference for changes in body weight over six months (Figure 2). The orlistat group had a 2 kg greater weight loss on average compared to the placebo group (month 6 group difference: −1.99, 95% CI: −3.81, −0.18) (Table 3). The orlistat group had more weight loss by three months, but compared to the placebo group, the difference was not statistically significant (group difference: −1.20, 95% CI: −2.57, 0.17). In the body weight models, adjusting for age, diet quality, and physical activity intensity, the estimates were similar (Model 2, Table 3).

### 3.4. Changes in other anthropometric measures

In addition to body weight, we measured waist circumference, body fat percentage, and muscle mass percentage. We observed no significant between-group differences for any of these measures over the study period. Within groups, body fat and muscle mass percentages showed minimal changes from baseline to six months. But there was a significant decrease in waist circumference in both study groups (Figure 2). Waist circumference reduced by an average of 4.15 cm (95% CI: −5.88, −2.43) in the orlistat group and 2.81 cm in the placebo group (95% CI: −4.51, −1.11).

### 3.5. Secondary outcome: changes in serum POP concentrations

For the secondary outcome analysis, we did not find statistically significant between-group differences for changes in serum POP concentrations. Further, we did not see a reduction in the POP concentrations over the six months. In the PBB-153 ln-transformed adjusted model, the between-group difference at month 6 was 0.136 (95% CI: −0.123, 0.395) (Table 3). Further adjustment for age, diet quality, and physical activity intensity did not vary the model estimates. In models adjusted for lipids, there were no significant differences between the study groups for any of the chemicals (Table 4). Although for lipid-standardized DDE concentrations, the orlistat group had a small increase at month 6 (mean change, orlistat group: 0.074, 95% CI: 0.018, 0.130).

### 3.6. Correlation analysis

When we explored the correlation between changes in body weight and changes in POP concentrations from baseline to six months, there was no correlation observed in the placebo group. In the orlistat group, there was no correlation for PBB-153 or PBDE-47 concentrations. But there was a weak negative correlation for total PCB or DDE concentrations, showing that as more weight was lost over the study period, exposure concentrations tended to increase (PCB: rho = −0.35, p=0.02; DDE: = −0.36, p=0.02).

### 3.7. Subgroup analysis

In the group with the highest initial POP concentrations (≥75^th^ percentile), there were no group differences or significant changes in the concentrations at six months (Table 5). This was also the case for those in the ≥5% weight loss group (placebo group, 29%; orlistat group, 41%). Although a statistically significant group difference was found for DDE exposure at month 3 (group difference: 0.186, 95% CI: 0.011, 0.361); where the placebo group saw a small decline in DDE concentrations (mean change, placebo group: −0.132, 95% CI: −0.265, 0.001) (Table 5).

### 3.8. Safety and adherence

Of the 100 enrolled participants, 80 experienced at least one AE (46% in the placebo group and 54% in the orlistat group), and out of the 154 AEs reported, three were serious. One was classified as “probably not related” and the other two were unrelated. All three serious AEs that occurred were among those receiving the placebo. Using a 5% threshold, non-serious AEs (categorized as gastrointestinal disorders, infections/infestations, or general disorders) were reported by 28 participants in the placebo group and 41 in the orlistat group. The most common gastrointestinal disorder was diarrhea and frequent or oily bowel movements, which were reported more in the orlistat group than in the placebo group (placebo group, 27%; orlistat group, 55%; p=0.004, Supplemental Table S4). For the remaining non-serious AEs, no statistically significant differences were observed between the study groups. Study protocol adherence was assessed by monitoring self-reported study medication use and completion of other study components. Over the six months, a higher proportion of participants in the placebo group (91%) consistently took their study medication than those in the orlistat group (75%) (p=0.04, Supplemental Table S3). Both study groups had high completion rates for physical activity and gastrointestinal symptom assessments (96%), while the dietary assessments had lower total completion rates (placebo group, 67%; orlistat group, 64%).

## 4. Discussion

This community-engaged trial investigated whether orlistat, a weight loss medication, could reduce lipophilic chemical levels in a community with high PBB exposure. The study was designed appropriately using blinding and stratification and was sufficiently powered to detect differences between the study groups. Although the orlistat group lost more weight, we did not observe a reduction in blood levels of PBB or the other persistent chemicals measured over the six-month study period. PBB levels are still elevated in this cohort compared to those in the general population (Chang et al., 2020). In this trial, 66% of participants had baseline PBB levels exceeding the national 95^th^ percentile (NHANES 2003–2004; 95^th^ percentile = 0.19 ng/mL) (National Center for Environmental Health, 2022).

The weight loss findings in our study are consistent with prior orlistat 60 mg dose studies (Anderson, 2007; Anderson et al., 2006; Smith et al., 2011; Thomas et al., 2011). To our knowledge, no other randomized controlled trial has explored the use of an orlistat intervention to reduce the body burden of persistent chemicals; however, there are a few studies of Olestra, a fat-substitute that was previously used in U.S. food products. In a town with residents exposed to high levels of PCBs, a small group of participants were provided with either chips containing Olestra (15g per day) or vegetable oil. Although limited by a small sample size (n=23), the one-year study found faster total PCB elimination rates in the Olestra group compared to the estimated elimination rates four to six years before the study. However, there was no significant difference in the change in PCB levels (total or PCB-153) from baseline between the groups (Jandacek et al., 2014). In addition, there was no difference in the DDE elimination rates between the groups. A sub-study of the Ole Study (Bray et al., 2002) included a group on a standard diet, a fat-reduced diet, and a fat-substituted diet with Olestra. Only plasma B-HCH concentrations differed significantly between the groups, with a decrease in the fat-substituted group. This was not seen for the other organochlorines measured, which included several PCB congeners. This study was limited by a small sample size (n=37) and a short three-month duration (Arguin et al., 2010).

Few studies have examined how other dietary interventions might influence the elimination of POPs. A small, two-month study of 15 women showed that vitamin C supplementation reduced the levels of PCBs and OCPs, but not PBDEs (Guo et al., 2016). Six months of fatty fish consumption did not alter the levels of organochlorines, dioxins, or non-dioxin PCBs when compared to a control group or a group consuming nuts (Dusanov et al., 2020). In a study comparing a vegetarian diet to a conventional diet, no differences were observed between the groups after 12 weeks for the POPs measured, except for PCB-169, where levels increased in the vegetarian diet group (Kahleova et al., 2016).

We hypothesized that orlistat might lower levels of persistent chemicals that are stored in fat, given that it has been shown to reduce body fat (compared to weight loss without orlistat). We implemented weight management strategies to encourage participants to increase their physical activity and modify their diets (such as reducing fat intake). Physical activity is suspected to enhance the mobilization and excretion of stored chemicals (Liu & Hou, 2023; Serrano et al., 2024). However, adipose tissue mobilization during weight loss facilitates the redistribution of POPs from fat stores to circulation (La Merrill et al., 2013). This release can lead to either stable or transiently elevated POP blood levels (Cheikh Rouhou et al., 2016; Lee et al., 2017; Moriceau et al., 2022). Prior research has shown that substantial weight loss achieved through bariatric surgery or calorie restriction, is associated with increased POP blood concentrations (Diaz-Gonzalez et al., 2024; Fenichel et al., 2021; reviewed in Jansen et al., 2017). These findings can be attributed to the temporary shifts in blood levels observed during a short follow-up period, as these chemicals are released from adipose tissue during major weight loss.

The weight loss totals we observed were considerably lower compared to the above-mentioned studies. The null findings we found for changes in serum POP concentrations are consistent with other dietary studies that reported lower weight loss totals (Arguin et al., 2010; Kahleova et al., 2016). Although in the orlistat group, total PCB and DDE concentrations showed an inverse relationship with weight change, a trend that was not observed in the placebo group or for the brominated compounds. The orlistat group lost more weight, which might explain a temporary rise in their serum concentrations. The results might also suggest that toxicokinetic differences between chemical classes influence how they are mobilized and redistributed in the body, subsequently altering serum concentrations (Moriceau et al., 2022).

In the main analysis, we modeled POP concentrations on a wet-weight basis. We also presented analyses where POP concentrations were standardized by lipids, which yielded similar results. Serum POP concentrations do not fully capture changes in the total body burden. Further, using either serum or lipid-adjusted concentrations can lead to an underestimation of the total body burden (Moriceau et al., 2022; Sontag et al., 2021). A study by Kim et al. (2011) found elevated blood levels following bariatric surgery, while the estimated total PCB body burden (based on adipose tissue concentration and total fat mass) decreased significantly over 6–12 months.

We were challenged in having to pivot the study partway through because of the COVID-19 pandemic. We could not conduct three-month study visits or blood draws, but participants completed other study assessments and self-reported their weight. However, we did not use self-reported weight data due to differences between the home and study scales. We implemented a statistical modeling strategy that was flexible enough to accommodate missing data. Some residual confounding from individual variability in diet and exercise modifications may not have been accounted for, but adjusting for these factors did not change the study findings. We observed most of the weight loss in the first three months; however, we could not account for individual toxicokinetic variations during this time. Additionally, we were limited by a short study duration of six months. Given the long biological half-lives of the chemicals we measured and temporary changes in POP blood levels during weight loss, it is difficult to observe their elimination over a short time period.

One of the notable strengths of the study is the lower attrition rates at three and six months (9% and 11%, respectively) when compared to other weight loss clinical trials (Fabricatore et al., 2009; Moroshko et al., 2011). This was influenced by motivated participants, access to study coaches, and guidance from a physician and nutritionist. The Michigan PBB Registry Leadership team, including Emory University researchers and several community partners, designed, planned, recruited, and promoted the study. Community partners provided resources that included identifying a local compounding pharmacy and local health department facilities for study appointments, and they assisted with facilitating research appointments during the COVID-19 pandemic. Data collected during the trial, including clinical tests, lipid measures, and anthropometric and dietary data, can be used to explore other study hypotheses.

This study was appropriately designed to determine the independent effect of orlistat on the study outcomes. Both study groups received the same weight management intervention (which included lowering dietary fat intake and increasing physical activity) following the FDA-approved recommendations for orlistat (FDA-Approved Weight Loss Aid ; U.S. Food and Drug Administration, 2015| Alli^®^). The findings of greater weight loss in the orlistat group were likely a result of the medication. Finally, participant group assignments were stratified by sex and BMI to account for these two main confounders.

POP concentrations remain detectable in both the general population and highly exposed populations (Chang et al., 2020; National Center for Environmental Health, 2022; Pumarega et al., 2016). Targeted interventions to reduce exposure, enhance elimination, and mitigate health consequences of POPs can result in substantial public health benefits. The present study found no reduction in serum POP concentrations after six months. However, it provides valuable insights regarding dietary interventions for weight loss and their impact on serum POP concentrations. The challenges in this research area have been previously documented (Cheikh Rouhou et al., 2016; Jandacek et al., 2024; Lee et al., 2017; Sargis et al., 2019). Long-term studies are needed to identify effective strategies for reducing the body burden of POPs. It is important to account for the mobilization of POPs during weight loss, as it impacts subsequent elimination from the body (Li & Wania, 2017). Further research is warranted to investigate the influence that temporary changes in circulating POP levels have on the total body burden and health outcomes.

## 5. Conclusions

This study began because the community wanted to identify a way to eliminate PBB from the body. It resulted from the shared dedication and commitment of stakeholders within the affected community. The six-month orlistat intervention did not decrease serum PBB or other POP concentrations. Further research is therefore needed to identify effective ways to decrease the body burden of this class of chemicals, which have long-term health impacts.

## Supplementary Material

supplementary material

Supplementary materials

Supplementary material associated with this article can be found, in the online version, at doi:10.1016/j.envadv.2026.100742.

## Figures and Tables

**Table 1:** Michigan PBB registry clinical trial inclusion and exclusion criteria.

Inclusion Criteria	Exclusion Criteria
• ≥18 years old • BMI ≥25 kg/m^2^ • Serum PBB level ≥1 ng/mL (from 2012–2015) or from a PBB exposed sub-group (e.g., lived on a PBB-quarantined farm, worked at the Velsicol chemical plant, family member of chemical plant worker, or lived in Michigan from 1973–1976) • Currently resided in Michigan • Able to engage in moderate physical activity (e.g., moderately paced walking)	Initial screening • Had type 1 diabetes • Had an organ transplant • Pregnant or lactating • Diagnosed problem absorbing food or having an eating disorder • History of bariatric surgery, pancreatitis, kidney stones, gallbladder disease or other serious chronic disease (e.g., congestive heart failure, chronic kidney disease) • Current use of any weight-loss medications, oral steroids, Coumadin, warfarin, or cyclosporine • Allergies to any ingredients of the orlistat OTC capsules Baseline Screening • Clinical blood test results: abnormal liver function or kidney function

**Table 2:** Michigan PBB registry clinical trial participant demographics, baseline characteristics, and POP concentrations.

	Placebo group N=45 N (%) or Median [IQR]	Orlistat group N=44 N (%) or Median [IQR]	Total N=89 N (%) or Median [IQR]

**Demographics**			
Sex			
Female	27 (60.0)	26 (59.1)	53 (59.6)
Male	18 (40.0)	18 (40.9)	36 (40.4)
Age, years	60.4 [54.0, 66.1]	60.7 [54.2, 67.0]	60.5 [54.0, 66.8]
Race			
White	42 (93.3)	42 (95.4)	84 (94.4)
More than one race	3 (6.7)	1 (2.3)	4 (4.5)
Unknown or missing	0 (0)	1 (2.3)	1 (1.1)
Ethnicity			
Not Hispanic or Latino	45 (100)	42 (95.4)	87 (97.8)
Hispanic or Latino	0 (0)	1 (2.3)	1 (1.1)
Unknown or missing	0 (0)	1 (2.3)	1 (1.1)
**Anthropometrics**			
BMI, kg/m^2^			
Overweight (25.0-29.9)	17 (37.8)	19 (43.2)	36 (40.4)
Obese (≥30)	28 (62.2)	25 (56.8)	53 (59.6)
Body Weight, kg	90.5 [77.8, 103.3]	88.7 [80.8, 104.9]	89.5 [80.1, 104.4]
Body Fat %	38.8 [34.1, 45.1]	40.4 [32.3, 46.8]	39.9 [33.0, 46.5]
Muscle Mass %	29.7 [27.6, 37.9]	29.8 [27.0, 37.8]	29.7 [27.3, 37.8]
Waist Circumference, cm	107.4 [98.4, 119.9]	106.1 [101.0, 116.6]	107.2 [99.3, 118.6]
**Lipid Profile**			
Total Cholesterol, mg/dL	187 [162, 217]	210.5 [184, 239]	200 [167, 231]
HDL, mg/dL	48 [41, 59]	58.5 [48.5, 71]	52 [42, 63]
LDL, mg/dL ^a^	108 [86, 134]	126.5 [95.5, 144]	116 [87, 137]
Triglycerides, mg/dL	166 [124, 234]	136.5 [115, 188.5]	150 [120, 213]
Total Lipids, mg/dL	682.3 [586.2, 776.8]	689.8 [590.3, 796.5]	682.3 [586.2, 786.0]
**Dietary Measures**			
Total Fat, %kcal	39.1 [33.5, 42.6]	36.9 [32.2, 42.2]	38.0 [32.6, 42.6]
Saturated Fat, %kcal	11.7 [10.4, 14.2]	13.0 [11.2, 14.5]	12.5 [10.5, 14.3]
Total Fiber, g/1000 kcal	8.4 [7.1, 12.9]	8.8 [7.3, 11.3]	8.7 [7.2, 11.8]
HEI-2015 Total Score	58.1 [51.0, 66.5]	54.8 [45.1, 61.1]	56.4 [46.5, 64.9]
**Physical Activity**			
Moderate Exercise			
None	22 (48.9)	12 (27.3)	34 (38.2)
<150 min/week	15 (33.3)	22 (50.0)	37 (41.6)
≥150 min/week	8 (17.8)	10 (22.7)	18 (20.2)
Strenuous Exercise			
None	36 (80.0)	29 (65.9)	65 (73.0)
<75 min/week	6 (13.3)	5 (11.4)	11 (12.4)
≥75 min/week	3 (6.7)	10 (22.7)	13 (14.6)
Total Physical Activity Score, MET-hours/ week	8.3 [3.5, 16.9]	13.5 [5.9, 34.5]	9.8 [4.6, 27.7]
**POP Concentrations**			
Wet weight (ng/mL)			
PBB-153	0.469 [0.156, 1.711]	0.260 [0.138, 0.767]	0.351 [0.145, 0.898]
PBDE-47	0.031 [0.015, 0.058]	0.029 [0.017, 0.053]	0.030 [0.016, 0.054]
ΣPCB_3_^b^	0.350 [0.182, 0.527]	0.252 [0.194, 0.467]	0.285 [0.191, 0.474]
DDE	0.549 [0.354, 0.844]	0.491 [0.340, 0.752]	0.518 [0.344, 0.841]
Lipid-adjusted (ng/g lipid)			
PBB-153	68.51 [20.91, 274.85]	36.12 [18.97, 104.72]	58.14 [20.29, 119.64]
PBDE-47	4.53 [2.18, 8.98]	3.85 [2.34, 7.51]	4.08 [2.24, 8.00]
ΣPCB_3_^b^	46.7 [31.45, 67.85]	41.35 [25.92, 58.87]	43.12 [28.61, 65.48]
DDE	80.41 [47.46, 136.37]	66.03 [49.00, 98.28]	72.2 [48.70, 116.61]

a LDL cholesterol not calculated when triglyceride levels >400 mg/dL (n=7).

b ΣPCB_3_ is the sum of three congeners 138, 153, and 180.

**Table 3:** Changes in study outcomes from baseline to 6 months in Michigan PBB Registry Clinical Trial participants ^a^.

Outcome	Placebo Mean Change (95% CI)	Orlistat Mean Change (95% CI)	Group Difference (Orlistat vs. Placebo) (95% CI) P-value ^b^	

Weight (kg)				
Model 1				
Month 3	−1.93 (−2.89, −0.97)*	−3.12 (−4.09, −2.16)*	−1.20 (−2.57, 0.17)	0.09
Month 6	−2.11 (−3.38, −0.83)*	−4.10 (−5.39, −2.81)*	−1.99 (−3.81, −0.18)	0.03
Model 2				
Month 3	−1.67 (−2.69, −0.64)*	−3.00 (−4.00, −2.01)*	−1.34 (−2.74, 0.06)	0.06
Month 6	−1.92 (−3.19, −0.65)*	−3.90 (−5.17, −2.63)*	−1.98 (−3.77, −0.19)	0.03
Ln(PBB-153 ng/mL)				
Model 1				
Month 3	−0.155 (−0.349, 0.039)	−0.064 (−0.258, 0.129)	0.091 (−0.185, 0.366)	0.51
Month 6	−0.138 (−0.319, 0.043)	−0.002 (−0.185, 0.181)	0.136 (−0.123, 0.395)	0.30
Model 2				
Month 3	−0.101 (−0.285, 0.084)	−0.003 (−0.182, 0.177)	0.098 (−0.155, 0.351)	0.44
Month 6	−0.082 (−0.275, 0.112)	−0.041 (−0.233, 0.151)	0.041 (−0.230, 0.312)	0.77
Ln(PBDE-47 ng/mL)				
Model 1				
Month 3	−0.018 (−0.206, 0.169)	−0.227 (−0.413, −0.041)*	−0.209 (−0.473, 0.056)	0.12
Month 6	−0.084 (−0.250, 0.083)	−0.136 (−0.304, 0.032)	−0.052 (−0.289, 0.185)	0.66
Model 2				
Month 3	−0.069 (−0.274, 0.135)	−0.169 (−0.374, 0.036)	−0.100 (−0.385, 0.185)	0.49
Month 6	−0.152 (−0.331, 0.026)	−0.132 (−0.313, 0.050)	0.021 (−0.23, 0.271)	0.87
Ln(ΣPCB3 ng/mL) ^c^				
Model1				
Month 3	−0.067 (−0.137, 0.004)	0.005 (−0.065, 0.075)	0.072 (−0.027, 0.171)	0.15
Month 6	−0.041 (−0.119, 0.036)	0.033 (−0.045, 0.111)	0.074 (−0.034, 0.183)	0.18
Model 2				
Month 3	−0.088 (−0.167, −0.009)*	−0.003 (−0.081, 0.076)	0.085 (−0.023, 0.193)	0.12
Month 6	−0.047 (−0.120, 0.026)	0.001 (−0.073, 0.075)	0.048 (−0.053, 0.148)	0.35
Ln(DDE ng/mL)				
Model 1				
Month 3	−0.055 (−0.124, 0.014)	−0.001 (−0.070, 0.067)	0.053 (−0.044, 0.151)	0.28
Month 6	−0.037 (−0.110, 0.036)	0.043 (−0.031, 0.118)	0.080 (−0.024, 0.185)	0.13
Model 2				
Month 3	−0.070 (−0.148, 0.008)	−0.001 (−0.078, 0.076)	0.069 (−0.039, 0.176)	0.21
Month 6	−0.038 (−0.108, 0.031)	0.011 (−0.059, 0.081)	0.049 (−0.046, 0.144)	0.31

a Constrained linear data analysis models: Model 1 adjusted for stratification variables (sex and BMI); Model 2 adjusted for stratification variables (sex and BMI), age, diet quality, and physical activity intensity. Estimates from Ln- transformed exposure models can be expressed as the percentage change (e^β^-1) x 100.

b p-value for the difference between study groups from baseline to 6 months.

c ΣPCB_3_ is the sum of three congeners 138, 153, and 180.

* Within-group changes from baseline to 6 months: p-value < 0.05.

**Table 4:** Changes in POP concentrations adjusted for lipids from baseline to 6 months in Michigan PBB Registry Clinical Trial participants^a^.

Outcome	Placebo Mean change (95% CI)	Orlistat Mean change (95% CI)	Group Difference (Orlistat vs. Placebo) (95% CI) P-value ^b^	

Ln(PBB-153)				
Wet weight (ng/mL)				
Month 3	−0.111 (−0.302, 0.079)	−0.010 (−0.207, 0.186)	0.101 (−0.171, 0.373)	0.46
Month 6	−0.122 (−0.308, 0.063)	0.014 (−0.181, 0.209)	0.136 (−0.134, 0.406)	0.32
Lipid-standardized (ng/g lipid)				
Month 3	−0.099 (−0.290, 0.091)	−0.001 (−0.198, 0.196)	0.098 (−0.177, 0.373)	0.48
Month 6	−0.105 (−0.290, 0.080)	0.032 (−0.162, 0.226)	0.138 (−0.132, 0.407)	0.31
Ln(PBDE-47)				
Wet weight (ng/mL)				
Month 3	0.023 (−0.166, 0.212)	−0.205 (−0.399, −0.011)*	−0.228 (−0.497, 0.041)	0.10
Month 6	−0.075 (−0.243, 0.094)	−0.147 (−0.325, 0.031)	−0.072 (−0.317, 0.173)	0.56
Lipid-standardized (ng/g lipid)				
Month 3	0.040 (−0.144, 0.224)	−0.188 (−0.377, 0.001)	−0.228 (−0.492, 0.036)	0.09
Month 6	−0.055 (−0.223, 0.114)	−0.130 (−0.306, 0.047)	−0.075 (−0.319, 0.169)	0.54
Ln(ΣPCB_3_) ^c^				
Wet weight (ng/mL)				
Month 3	−0.006 (−0.053, 0.041)	0.056 (0.008, 0.104)*	0.062 (−0.005, 0.128)	0.07
Month 6	−0.008 (−0.066, 0.049)	0.043 (−0.017, 0.104)	0.052 (−0.031, 0.135)	0.22
Lipid-standardized (ng/g lipid)				
Month 3	0.002 (−0.047, 0.050)	0.071 (0.022, 0.120)*	0.069 (−0.00001, 0.139)	0.05
Month 6	0.001 (−0.058, 0.059)	0.059 (−0.002, 0.120)	0.058 (−0.026, 0.143)	0.18
Ln(DDE)				
Wet weight (ng/mL)				
Month 3	0.001 (−0.045, 0.048)	0.045 (−0.002, 0.092)	0.044 (−0.021, 0.109)	0.18
Month 6	−0.008 (−0.060, 0.045)	0.055 (−0.0003, 0.110)	0.063 (−0.013, 0.138)	0.10
Lipid-standardized (ng/g lipid)				
Month 3	0.011 (−0.038, 0.060)	0.062 (0.012, 0.112)*	0.051 (−0.019, 0.121)	0.15
Month 6	0.001 (−0.053, 0.054)	0.074 (0.018, 0.130)*	0.074 (−0.004, 0.151)	0.06

a Constrained linear data analysis models: Wet-weight models adjusted for stratification variables (sex and BMI) and total serum lipids; lipid-standardized models adjusted for stratification variables (sex and BMI). Estimates from Ln-transformed exposure models can be expressed as the percentage change (e^β^-1) x 100.

b p-value for the difference between study groups from baseline to 6 months.

c ΣPCB_3_ is the sum of three congeners 138, 153, and 180.

* Within-group changes from baseline to 6 months: p-value < 0.05.

**Table 5:** Changes in POP concentrations from baseline to 6 months among two sub-groups: a) Baseline POP concentrations ≥75^th^ percentile; b) ≥5% weight loss ^a^.

Outcome	Placebo Mean change (95% CI)	Orlistat Mean change (95% CI)	Group Difference (Orlistat vs. Placebo) (95% CI) P-value ^b^	

a) Baseline level ≥75^th^ percentile				
Ln(PBB-153 ng/mL)				
Month 3	−0.041 (−0.209, 0.126)	0.030 (−0.138, 0.199)	0.071 (−0.169, 0.312)	0.54
Month 6	−0.046 (−0.179, 0.086)	0.022 (−0.116, 0.160)	0.068 (−0.123, 0.259)	0.46
Ln(PBDE-47 ng/mL)				
Month 3	−0.180 (−0.730, 0.369)	−0.463 (−1.025, 0.099)	−0.283 (−1.082, 0.516)	0.47
Month 6	−0.183 (−0.520, 0.154)	−0.164 (−0.517, 0.189)	0.019 (−0.480, 0.518)	0.94
Ln(ΣPCB_3_ ng/mL) ^c^				
Month 3	−0.158 (−0.346, 0.031)	−0.057 (−0.242, 0.128)	0.101 (−0.168, 0.369)	0.44
Month 6	−0.163 (−0.360, 0.035)	−0.098 (−0.304, 0.108)	0.065 (−0.223, 0.352)	0.64
Ln(DDE ng/ mL)				
Month 3	−0.002 (−0.167, 0.164)	0.054 (−0.112, 0.221)	0.056 (−0.180, 0.292)	0.62
Month 6	0.044 (−0.164, 0.252)	−0.016 (−0.233, 0.200)	−0.060 (−0.356, 0.236)	0.68
b) ≥5% weight loss				
Ln(PBB-153 ng/mL)				
Month 3	−0.183 (−0.465, 0.099)	0.037 (−0.203, 0.277)	0.220 (−0.150, 0.591)	0.22
Month 6	0.003 (−0.393, 0.399)	−0.027 (−0.366, 0.311)	−0.030 (−0.545, 0.485)	0.91
Ln(PBDE-47 ng/mL)				
Month 3	−0.023 (−0.264, 0.218)	−0.112 (−0.316, 0.091)	−0.089 (−0.408, 0.231)	0.57
Month 6	−0.010 (−0.203, 0.183)	−0.009 (−0.172, 0.154)	0.001 (−0.256, 0.258)	0.99
Ln(ΣPCB_3_ ng/mL) ^c^				
Month 3	−0.099 (−0.229, 0.031)	0.032 (−0.078, 0.142)	0.131 (−0.041, 0.303)	0.13
Month 6	−0.029 (−0.196, 0.137)	0.110 (−0.031, 0.251)	0.139 (−0.081, 0.359)	0.21
Ln(DDE ng/ mL)				
Month 3	−0.132 (−0.265, 0.001)	0.054 (−0.059, 0.166)	0.186 (0.011, 0.361)	0.04
Month 6	−0.022 (−0.184, 0.141)	0.140 (0.003, 0.278)	0.162 (−0.053, 0.377)	0.13

a Constrained linear data analysis models adjusted for stratification variables (sex and BMI). Estimates from Ln-transformed exposure models can be expressed as the percentage change (e^β^-1) x 100.

b p-value for the difference between study groups from baseline to 6 months.

c ΣPCB_3_ is the sum of three congeners 138, 153, and 180.

## Data Availability

The data generated for this study is not publicly available and the consent form language restricts access to the data. Contact the Corresponding Author about data availability through a formal request and data use agreement with the Michigan PBB Registry and Emory University.

## Abbreviations:

PBB: Polybrominated biphenyl
POP: Persistent organic pollutant
PCB: Polychlorinated biphenyl
PBDE: Polybrominated diphenyl ether
DDT: Dichlorodiphenyltrichloroethane
DDE: Dichlorodiphenyldichloroethylene
OCP: Organochlorine pesticide
B-HCH: B-hexachlorocyclohexane
NHANES: National health and nutrition examination survey
OTC: Over-the-counter
FDA: Food and drug administration
eGFR: Estimated glomerular filtration rate
ALT: Alanine aminotransferase
TSH: Thyroid-stimulating hormone
BMI: Body mass index
SST: Serum-separator tube
LOD: Limit of detection
ASA24: Automated self-administered 24-hour recall
HEI-2015: Healthy eating index
PAQ: Physical activity questionnaire
AE: Adverse event
GST: Gastrointestinal symptom tracker
cLDA: Constrained linear data analysis models
CI: Confidence interval
