# Supplementary material for: A community-engaged randomized controlled trial assessing the effectiveness of a weight loss aid in reducing the body burden of persistent organic pollutants
Source: Environ Adv. Author manuscript; Available in PMC 2026 Sep 17. (PMC13576412; doi:10.1016/j.envadv.2026.100742)
Supplement: Supplementary file 1 [file NIHMS2203475-supplement-supplementary_material.docx]

**Supplementary Tables**

**Table S1**. Ingredient lists

| Orlistat | |
| --- | --- |
| Active: | Orlistat (60 mg) |
| Inactive: | FD&C Blue No. 2, edible ink, gelatin, iron oxide, microcrystalline cellulose, povidone, sodium lauryl sulfate, sodium starch glycolate, talc, titanium dioxide |
| Multivitamin | |
| Active: | Vitamin A (5000 IU), Vitamin C (90 mg), Vitamin D-3 (400 IU),  Vitamin E (30 IU), Vitamin B-1 (3 mg), Vitamin B-2 (3.4 mg), Niacin (20 mg), Vitamin B-6 (3 mg), Folic Acid (400 mcg), Vitamin B-12 (9 mcg), Biotin (30 mcg), Pantothenic Acid (10 mg), Calcium (45 mg), Phosphorus (35 mg) |
| Inactive: | Microcrystalline Cellulose, Gelatin, Stearic Acid, Hypromellose,  Croscarmellose Sodium, Magnesium Stearate, Magnesium Silicate, Silica, FD&C Red #40 Lake, Polyvinylpyrrolidone, Polyethylene Glycol, Mineral Oil, Titanium Dioxide and FD&C Yellow #6 Lake |

**Table S2.** Michigan PBB Registry Clinical Trial study components by time period

|  | Time of Assessment | | | | |
| --- | --- | --- | --- | --- | --- |
|  | Screening | | Study Period | | |
| Study Components | Initial | Baseline | Enrollment | Month 3 | Month 6 |
| Screening questionnaire | X |  |  |  |  |
| Health history questionnaire | X |  |  |  |  |
| Blood draws |  |  |  |  |  |
| Serum POP concentrations |  | X |  | X | X |
| Clinical function blood tests |  | X |  |  | X |
| Lipid panel |  | X |  | X | X |
| Anthropometric Measurements |  | X |  | X | X |
| Randomization |  |  | X |  |  |
| Dietary Assessments |  |  | X | X | X |
| Physical Activity Assessments |  |  | X | X | X |
| Gastrointestinal Symptom Assessments |  |  | X | X | X |
| Pill count |  |  |  | X | X |
| Compensation |  |  |  | $40 | $60 |

**Table S3.** Michigan PBB Registry Clinical Trial Study adherence for the 6-month study

| Study Component ^a^ | Placebo group N=45  N (%) | Orlistat group  N=44  N (%) |
| --- | --- | --- |
|  |  |  |
| Study medication ^b^ |  |  |
| <80% taken | 4 (8.9) | 11 (25.0) |
| ≥80% taken | 41 (91.1) | 33 (75.0) |
| Dietary Assessments |  |  |
| Partial completion | 15 (33.3) | 16 (36.4) |
| Total completion | 30 (66.7) | 28 (63.6) |
| Physical Activity Assessments |  |  |
| Partial completion | 2 (4.4) | 2 (4.6) |
| Total completion | 43 (95.6) | 42 (95.5) |
| Gastrointestinal Symptom Assessments |  |  |
| Partial completion | 2 (4.4) | 2 (4.6) |
| Total completion | 43 (95.6) | 42 (95.5) |

^a^ Completion of assessments defined as: dietary (total, 5 completed; partial, 1-4 completed),

physical activity (total, 3 completed; partial, 1-2 completed), gastrointestinal symptoms

(total, 3 completed, partial; 1-2 completed)

^b^ Statistically significant difference between study groups (p<0.05)

**Table S4.** Incidence of non-serious adverse events reported by Michigan PBB Registry

Clinical Trial participants (n=100, enrolled and randomized to a study group)

| Organ System ^a^ | Placebo group  N=49  N (%) | Orlistat group  N=51  N (%) |
| --- | --- | --- |
|  |  |  |
| Gastrointestinal Disorders |  |  |
| Diarrhea and frequent or oily bowel  Movements ^b^ | 13 (26.5) | 28 (54.9) |
| Stomach ache or gassy | 5 (10.2) | 1 (2.0) |
| General Disorders |  |  |
| Headaches | 5 (10.2) | 6 (11.8) |
| Fatigue | 0 (0) | 3 (5.9) |
| Dizziness | 0 (0) | 3 (5.9) |
| Falls | 1 (2) | 3 (5.9) |
| Infections and Infestations |  |  |
| COVID-19 | 3 (6.1) | 2 (3.9) |
| Cold/flu/respiratory symptoms | 12 (24.5) | 14 (27.5) |

^a^ Includes non-serious adverse events that occurred in more than 5% of one study group

^b^ Statistically significant difference between study groups (p<0.05)
